# Inclusive Marketing in Ophthalmology: building the consumers’ experiences

**DOI:** 10.22336/rjo.2025.01

**Published:** 2025

**Authors:** Consuela-Mădălina Gheorghe

According to Kotler and Armstrong, the primary aim of marketing is to sell and generate a profit, implying the need for efficient targeting and product positioning (Kotler P. & Armstrong G. Principles of Marketing. 17th ed., 2018, Pearson, U.K.). However, a shift from product to consumer has occurred in the last 20 years, coinciding with the development of technology, which has led marketers to cater to increasingly diverse consumer requirements, even in the ophthalmology field.

It has become increasingly evident that marketing is a powerful tool that enables brands to connect with consumers, thereby enriching their experiences and fostering strong relationships. Moreover, consumers expect brands to be socially responsible.

Regarding the social field, one of the most important subjects is inclusivity. According to the World Health Organization, 15% of the world’s population lives with a disability (Shalvi M. Inclusive Marketing: Building a Loyal Customer Base Through Accessible Digital and Physical Experiences. Digital Marketing. Boston Hospitality Review. July 2022). At the same time, this percentage is often underrepresented and overlooked in marketing efforts (Shalvi M. Inclusive Marketing: Building a Loyal Customer Base Through Accessible Digital and Physical Experiences. Digital Marketing. Boston Hospitality Review, July 2022).

To make marketing more inclusive, health care and ophthalmological organizations need to be more creative and go beyond the required regulations, engaging directly with diverse consumer segments.

Additionally, it can be stated that ophthalmological consumers need to feel a sense of belonging or identify with the brand or organization to become loyal consumers. A relevant example is the consumers of a specific eyeglasses or sunglasses brand.

Inclusive marketing is the process of creating campaigns and initiatives that “enable marginalized or underrepresented groups to experience and connect with brands fully” (Shalvi M. Inclusive Marketing: Building a Loyal Customer Base Through Accessible Digital and Physical Experiences. Digital Marketing. Boston Hospitality Review. July 2022). It elevates underrepresented voices and can influence positive social change. With Diversity, Equity, and Inclusion (DEI) being a core value for many companies, it is expected that brands will follow through on such promises (Shalvi M. Inclusive Marketing: Building a Loyal Customer Base Through Accessible Digital and Physical Experiences. Digital Marketing. Boston Hospitality Review, July 2022). DEI is a term that refers to equality and access to resources for all individuals, irrespective of their gender, race, religion, ethnicity, or sexual identity. This is particularly important in ophthalmology, as disparities often exist due to racial and social factors; therefore, promoting inclusive policies in the field is vital. However, in this case, ophthalmological marketing campaigns should align with cultural sensitivities, avoid perpetuating stereotypes, and ensure resonance with the target consumers.

Disabilities are of different kinds. The Center for Disease Control (CDC) highlights six types: Mobility, Cognition, Independent Living, Hearing, Vision, and self-care. The World Health Organization (WHO) estimated that by 2050, about 2.5 billion people will have some degree of hearing loss. The American Foundation for the Blind found that over 32 million American adults “reported experiencing vision loss”. According to Special Olympics, approximately 200 million people have an intellectual disability, and more than 12% of individuals who contracted COVID-19 have an olfactory dysfunction that prevents them from having a permanent ability to smell. The lack of these abilities or the presence of these disabilities make it harder for the individuals to be able to build or form experiences regarding the use of products or services, because experiences are based on the five human senses (Shalvi M. Inclusive Marketing: Building a Loyal Customer Base Through Accessible Digital and Physical Experiences. Digital Marketing. Boston Hospitality Review. July 2022).

The best practices for integrating consumers with disabilities appear to be primarily in the digital field. Digital practices aim to raise awareness and ensure the best interaction between the consumer and the website's content, thereby enhancing the connection with the product or service.

Regarding physical experiences, individuals with sensory impairment can rely on other existing senses to have an experience. For example, if an individual has a visual impairment, their hearing, taste, smell, and touch abilities will be enhanced and utilized to provide them with the best and most complete experience of a product or service that would have been more effectively perceived visually. This is typically considered the individual’s ability to adapt to new living conditions.

There are specific elements that integrate more than one sense into traditional marketing techniques and allow the inclusion of individuals with impairments: sight, sound, smell, touch, and taste.

As far as sight is concerned, nowadays, many marketing messages are transmitted, and communication is established through videos, texts, images, and other visual media. These elements can be easily used to explain sound, smell, touch, and taste. Moreover, color can be used to describe facial expressions for people with cognitive impairments.

Sound is used in conjunction with visual elements to provide consumers with a comprehensive experience of a product or service.

Smell triggers the consumers’ emotions, enabling them to remember a positive or negative experience. In ophthalmology marketing, smell is rarely used.

Touch is the sense related to the ability to identify textures, quality, shapes, weights, and other physical properties. In ophthalmology, the Braille messages or guides are often used with the aim of inclusivity.

Taste is the most challenging to describe due to its subjectivity. Thus, to satisfy the needs and desires of consumers, the products should be customized for each individual or group of individuals. Therefore, for a blind person, whose sense of sight is nonexistent, a product customized to his taste and smell would be a proof of inclusivity.

In conclusion, inclusive marketing is essential in ophthalmology, as it is in other fields, because it entails creativity, higher costs (due to the creation of customized products or services), and increased accessibility of consumers to products or services through the digital field.

**Assist. Prof. Consuela-Mădălina Gheorghe, PhD, Philologist, Certified translator**

